# Artificial intelligence-driven microbiome data analysis for estimation of postmortem interval and crime location

**DOI:** 10.3389/fmicb.2024.1334703

**Published:** 2024-01-19

**Authors:** Ze Wu, Yaoxing Guo, Miren Hayakawa, Wei Yang, Yansong Lu, Jingyi Ma, Linghui Li, Chuntao Li, Yingchun Liu, Jun Niu

**Affiliations:** ^1^Department of Dermatology, General Hospital of Northern Theater Command, Shenyang, China; ^2^Department of Dermatology, The First Hospital of China Medical University, Shenyang, China; ^3^Key Laboratory of Immunodermatology, Ministry of Education and NHC, Shenyang, China; ^4^National Joint Engineering Research Center for Theranostics of Immunological Skin Diseases, Shenyang, China; ^5^Beijing Anzhen Hospital, Capital Medical University, Beijing, China

**Keywords:** forensic microbiology, artificial intelligence, microbiome, postmortem interval, crime location

## Abstract

Microbial communities, demonstrating dynamic changes in cadavers and the surroundings, provide invaluable insights for forensic investigations. Conventional methodologies for microbiome sequencing data analysis face obstacles due to subjectivity and inefficiency. Artificial Intelligence (AI) presents an efficient and accurate tool, with the ability to autonomously process and analyze high-throughput data, and assimilate multi-omics data, encompassing metagenomics, transcriptomics, and proteomics. This facilitates accurate and efficient estimation of the postmortem interval (PMI), detection of crime location, and elucidation of microbial functionalities. This review presents an overview of microorganisms from cadavers and crime scenes, emphasizes the importance of microbiome, and summarizes the application of AI in high-throughput microbiome data processing in forensic microbiology.

## Introduction

Microorganisms, both from cadavers and their surroundings, experience microbial succession, which refers to the sequential and orderly change of microbial communities in a particular environment over time (Díez López et al., 2022). This has become particularly pivotal in forensic investigations, providing insights relevant to the decomposition process, postmortem interval, cause of death, and other essential aspects (Ventura Spagnolo et al., 2019). As advancements in bioinformatics and high-throughput sequencing technologies arise, leveraging these microbial communities is increasingly vital. However, the vastness and complexity of this data introduce significant challenges (Liao et al., 2023; Dobay et al., 2019), making the incorporation of AI methodologies indispensable for efficient and precise analysis (Rajkomar et al., 2019; He et al., 2022). This review delves into the landscape of forensic investigation in the context of microbiome data, emphasizing the transformative role of AI in data interpretation and the potential challenges to address.

## Microbial succession during decomposition

Microbial succession refers to the changes in microbial populations on and around a corpse over time. Different microbial species become dominant at various stages of decomposition, reflecting alterations in the available resources and environmental conditions (Deel et al., 2021).

1n 1965, Payne (1965) identified several stages of decomposition in carrion which is determined by specific physical conditions and different types of insect colonization, provided a framework for understanding of microbial succession in cadavers. The fresh stage of decomposition begins with indigenous microorganisms covering the skin, oral cavity, gastrointestinal tract and other parts of the body, begin to multiply and spread, such as *Escherichia coli* (Metcalf et al., 2016; Speruda et al., 2022). Researchers (Adserias-Garriga et al., 2017b) analyzed the oral swab samples from three corpses and identified *Firmicutes* and *Actinobacteria* are the predominant phyla in the fresh stage. As decomposition progresses, the accumulation of gasses due to microbial metabolism causes the cadaver to bloat. *Clostridium* species proliferate, participating in fermentative decomposition processes (Hyde et al., 2013). In the active decay stage, leakage of decomposition fluids results in the formation of a cadaver decomposition island (CDI) referring to a localized area surrounding and under a decomposing carcass. Anaerobic conditions inside the cadaver change to become more aerobic as body cavities burst and allow air to enter. *Bacillus* and *Pseudomonas* species are bacteria commonly found at this stage (Pechal et al., 2014; Dash and Das, 2022). As decay advanced, *Acinetobacter*, *Sphingomonas*, and other bacteria adept at surviving in lower-nutrient conditions may become more prominent. Fungi may become more prevalent as the environment becomes less suitable for bacterial growth. Aspergillus, Penicillium, and Candida are dominant in the bloated and putrefaction stage (Sidrim et al., 2010). The microbial profile in this stage can be dominated by soil bacteria, such as *Pseudomonads* and other organisms that are capable of surviving in a nutrient-depleted environment (Carter et al., 2007). Toward the skeletonization stage, remaining soft tissues are largely depleted, the microbiome largely consists of bacteria and fungi in CDI. External microbes can interact with the cadaver’s native microbes, leading to potential symbiotic relationships, competitive interactions, or even the suppression of certain species. Environmental factors, like soil type and moisture, influence the microbial communities on and within the remains (Adserias-Garriga et al., 2017a; Taylor et al., 2023). The precise microbial succession can depend heavily on environmental, geographical, and individual factors.

## The importance of microbiome in forensic microbiology and the limitations of its traditional analysis

The human postmortem microbiome, including the thanatomicrobiome (the microbiome of blood, fluids and internal organs of cadavers) and epinecrotic microbial communities (the microbiome on surfaces of decaying remains), plays an important role in forensic investigations (Javan et al., 2016a). Due to the ease of sample acquisition, most research has focused on the latter, especially in gastrointestinal tract, the oral cavity, and skin (Dash and Das, 2022). Hyde et al. (2013) explored the microbiome alterations from aerobic to anaerobic bacteria during the bloat stage of decomposition by employing 16S rRNA gene pyrosequencing to analyze bacterial samples. Metcalf et al. (2013) explored the microbiome extracted from cadavers and their surroundings in the process of decomposition to analyze 16S and 18S rRNA sequences. The study from Pechal (Pechal et al., 2014) focused on bacterial communities extracted from buccal cavity and skin during the decomposition process, analyzed via 16S rRNA gene pyrosequencing and delved into analyzing the relative abundance of bacterial taxa in different sampling regions, studying the variation in bacterial communities at both phylum and family taxonomic levels over a measured physiological time.

However, analyzing microbiome data requires specialized bioinformatics expertise, indicating that not all forensic laboratories possess the capability to undertake such evaluations. Numerous factors, such as sample processing methods and sequencing technologies, can influence microbial abundance, even though high-throughput sequencing technology is advanced, it can sometimes miss microorganisms in low abundance or misidentify certain species (Schloss et al., 2009; Johnson et al., 2019). During the processes of sample extraction and processing, there’s the possible introduction of external microbial contamination, which can distort analytical results and lead to misunderstandings (Salter et al., 2014). Additionally, microbial communities comprising hundreds to thousands of species, complicate the analysis process. A myriad of analytical methods and statistical models, such as random forest models, generalized additive models, and generalized linear models, needed to be selected approximately to identify significant taxa, constructing predictive models using the change of bacterial community composition during the decomposition of cadavers require meticulous execution and interpretation. Handling and making sense of datasets, ensuring the precision of the alignment and classification of sequences, and scrutinizing potential taxonomic, spatial, and temporal variability in the data also pose significant challenges (Metcalf et al., 2013; Pechal et al., 2014).

In conclusion, while microbiome data offers unparalleled insights in forensic investigation, the present methods used for its analysis need continual improvement.

## Application of AI in high-throughput microbiome data processing in forensic microbiology

As modern high-throughput sequencing technology advances, the volume of macrobial data has overtaken the processing capabilities of traditional techniques. AI comes forward as solutions, particularly in forensic microbiology, where these technologies assist in deriving meaningful patterns from extensive datasets (Yuan et al., 2023).

Quality control for raw microbiome data is paramount for subsequent analysis. AI techniques, such as deep learning, facilitate automatic detection and correction of sequencing errors, removal of low-quality data, and filtration of potential contaminant sequences (Knights et al., 2011b; Borgman et al., 2022). Researchers integrates deep regression forests (DRFs) with convolutional neural networks (CNN) to improve the robustness against erroneous data entry and extreme population heterogeneity (Nolte et al., 2023). Traditional alignment-based methods can be inefficient with vast datasets. AI-driven models can efficiently identify and classify microbial sequences, crafting a detailed species abundance profile for every sample (Fiannaca et al., 2018; Reiman et al., 2021). Machine learning models can forecast the functional potential of microbial communities, suggesting possible impacts on hosts and environments. For instance, random forests or support vector machines can predict microbial functional pathways, giving forensic indications about the deceased’s lifestyle, health, or environmental exposures (Wang M. G. et al., 2022). Deep learning models, such as CNNs, allow researchers to identify specific biomarkers from microbial data. This could include microbial communities or gene expression patterns linked to specific toxin exposures (Liu et al., 2023). Microorganism that is stably expressed in different postmortem organs can be used as a biomarker(Javan et al., 2016b), and further, a series of dynamically changing biomarkers can be combined to accurately predict PMI. A random forest regression model based on 18 important genera obtained minimal cross-validation error demonstrated good predictive performance with a mean absolute error (MAE) of 1.27 ± 0.18 day within 36 day of the decomposition process (Cui et al., 2022). For forensic investigations, machine learning algorithms based on microbial succession can be combined with classification and regression models to accurately predict postmortem interval, the authors conducted OTU clustering and taxonomic annotation on the sequencing data of epinecrotic communities from cecal feces of rats and humans within 30 days after death, and applied the recursive feature elimination (RFE) with random forests algorithm to find the most effective feature subset. Subsequently, a double-layer model for PMI prediction was established, which discriminated groups of PMI 0–7d and 9–30d using random forest (RF), support vector machine (SVM), multi-layer perceptron (MLP), and logistic regression (LR) methods, and then the RF regression model was conducted to effectively predict PMI (Li N. et al., 2023), the researchers (Wang J. et al., 2022) studied the microRNAs and circular RNAs in blood samples and used various machine learning algorithms to conduct forensic age estimation, random forest regression model performed the best performance, multimarker approaches based on machine learning can make human identification using the skin microbiome more robust (Sherier et al., 2021).

**Figure 1:**
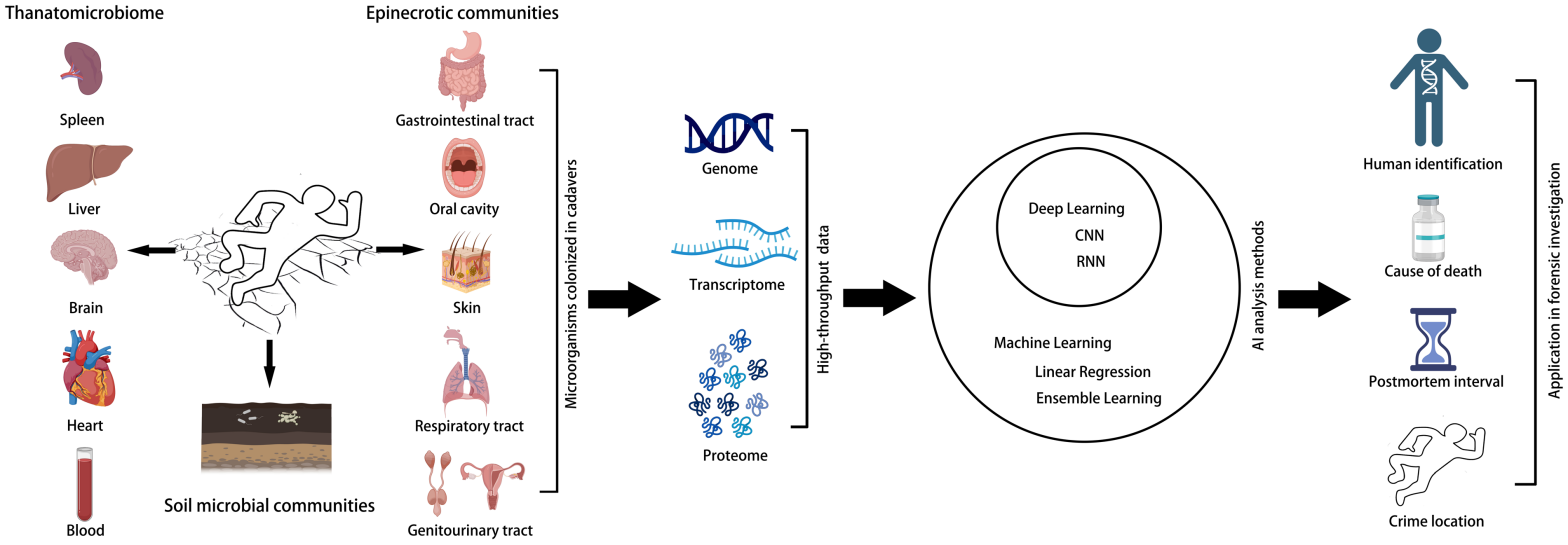
The application of AI in high-throughput microbiome data processing in forensic microbiology. The human postmortem microbiome and soil microbial communities are processed into high-throughput data for artificial intelligence analysis and ultimately used for forensic investigations.

In a forensic context, microbiological analysis is usually performed based on metagenomic sequencing, which is the most commonly used technique, but for samples such as hair or bone that DNA is absent or degraded, transcriptomic and proteomic approaches may be more applicable (Cláudia-Ferreira et al., 2023). However, the correlations and differences between diverse microorganisms after human death and their temporal changes, as well as the interactions with environmental factors, depict the dynamic changes in postmortem microbiome, the present approaches are not comprehensive. Multidimensional information is often needed to be combined to improve the prediction accuracy, for instance, the researchers integrated metabolomics, protein microarray electrophoresis, and Fourier transform-infrared spectroscopy data, and then analyzed using machine learning algorithms, improved the accuracy of PMI estimation, offered a panoramic view of the case (Li J. et al., 2023). The application of AI in high-throughput microbiome data processing in forensic microbiology is summarized in Figure 1.

## Artificial intelligence-based microbiome analysis for PMI estimation

PMI estimation is very important in forensic scenarios. Microbial succession, also known as the temporal changes of prominent microorganisms during different stages of decomposition, making up for the shortcomings of traditional methods that relying on physical processes after death, such as the drop in temperature of corpses and livor mortis, provides an important basis for predicting PMI (Deel et al., 2021). AI techniques have become the main analytical method for high-throughput microbiome data due to its efficiency and robustness in handling vast datasets, automatically identifying and correcting sequences, and allowing for the combination of multi-omics datasets (Fiannaca et al., 2018; Borgman et al., 2022; Li J. et al., 2023; Nolte et al., 2023).

**Figure 2:**
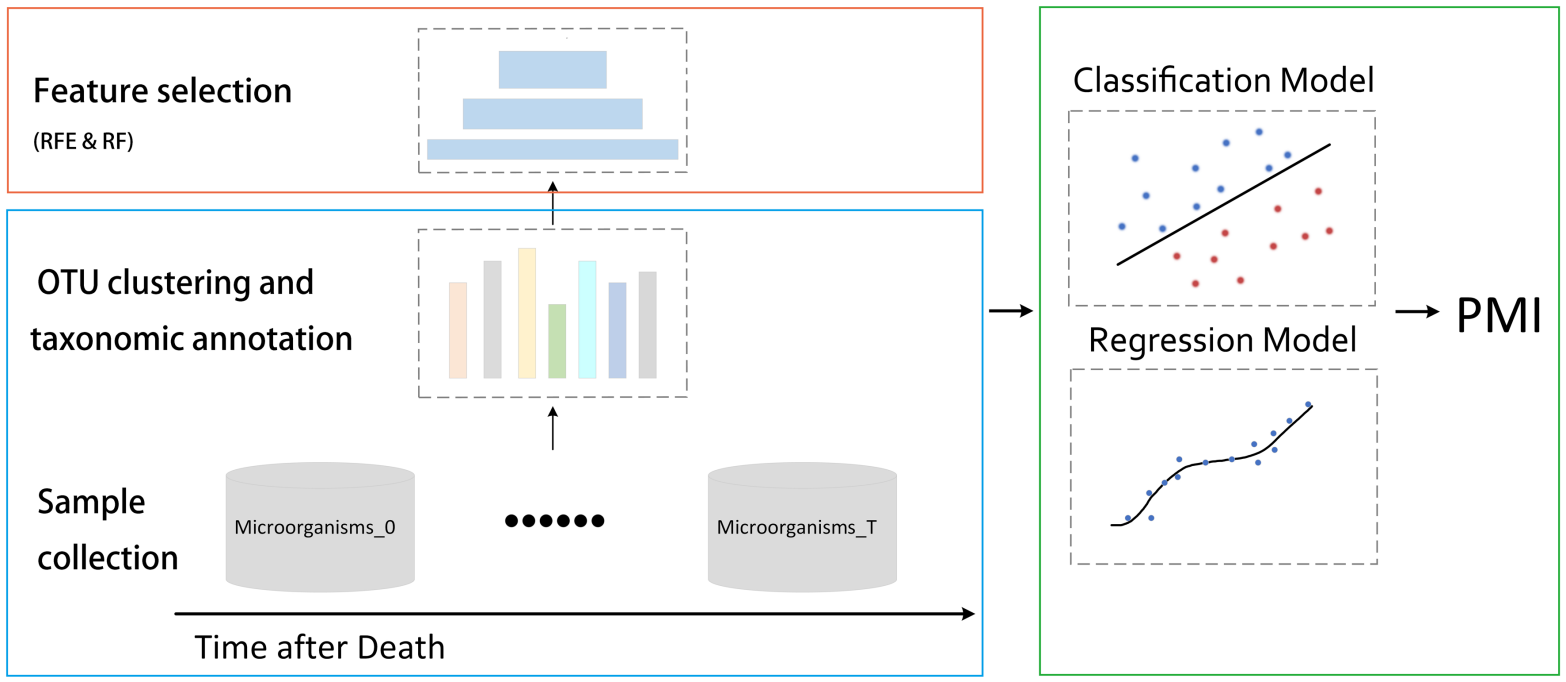
The process of artificial intelligence-based microbiome analysis for PMI estimation. Processing high-throughput data from human postmortem microbiome, after clustering and taxonomic annotation, selecting the best performed feature subset, and ultimately establishing a model for PMI estimation.

In the process of utilizing AI for PMI prediction, the first step involves conducting high-throughput sequencing for the corpse’s microorganisms. Given the inherent noise in sequencing data, rigorous quality control, filtration, and annotation are vital to ensure the robustness of model (Lee et al., 2022). Deep learning methodologies, including CNNs and recurrent neural networks (RNNs), have shown promise in analyzing these datasets. With CNNs excelling particularly in image processing, data representations like operational taxonomic units (OTUs) tables or microbial relative abundance heatmaps can be interpreted as “images” with appropriate transformation. Researchers used phylogenetic tree to represent spatial relationship, used OTU sorted by abundance as input to CNN, and developed machine regression model that integrates multiple spatially correlated kernels of microbial data with optimal weight sets to improve the predictive performance for health outcomes (Li B. et al., 2023). Some researchers have also used random forest, support vector machine, multilayer perceptron, and logistic regression methods based on the relative abundance of taxa at the genus level to establish robust classification model (Li N. et al., 2023). By conceptualizing corpse decomposition as a time-series event, RNNs are equipped to register the sequential changes in microbiome data. Researchers have achieved accurate prediction of key bacterial species and environmental factors through a RNN model based on dynamically changing microbial data (Thompson et al., 2023). These AI models, when trained appropriately, can identify patterns in microbiome data that correlate with PMI. The process of artificial intelligence-based microbiome analysis for PMI estimation is shown in Figure 2.

Researchers initiated the study with high-throughput sequencing data of bacterial communities in gravesoil sample associated with the decomposition of mouse cadavers, and identified temporal patterns of microbial communities, for example, the gradual increased abundance of *Proteobacteria* while the decrease of *Actinobacteria* during the decomposition, then utilized a random forest model to predict PMI within 36 days based on the microbial succession (Cui et al., 2022). RNNs are widely used in modeling microbial community dynamics due to their ability to capture complex biological behaviors associated with higher order interactions (Baranwal et al., 2022). The regression model that established by random forest algorithm based on intestinal microbial community succession of corpses exhibited satisfactory performance for PMI estimation (Liu et al., 2022; Zhang et al., 2022). The 16S rRNA sequences with the highest abundance in the clustered OTUs were taxonomically annotated, and abundance was visualized using non metric multidimensional scaling to characterize the temporal changes in the microbial community within 30 days during the decay of mouse corpse. There are significant differences in the composition of microbial communities, especially between the 0–7 day and 9–30 day decomposition stages. Therefore, a two-layer model for PMI prediction based on bacterial sequences data was developed by combining classification and regression models using machine learning algorithms (Li N. et al., 2023).

Performance of these models is typically validated using independent test sets, and when compared with traditional methods, AI models have demonstrated superior prediction accuracy (Baranwal et al., 2022; Cui et al., 2022; Liu et al., 2022; Zhang et al., 2022; Li N. et al., 2023). It is imperative that the efficacy of these models be validated under diverse conditions to ascertain their broad applicability. Once optimized, these models can efficiently and accurately process data from various forensic cases.

## AI in predicting crime location based on microbial data

Unlike geographical location that represent a “broader area”, the place of death specifically refers to the exact location where a person has died, and estimating the crime location is essential in forensic investigations. Researchers (Karadayı, 2021) have demonstrated by analyzing soil samples extracted from simulated murder scenes that microorganisms, especially fungal DNA allowed to identify evidence samples and crime scene samples at phylum and class and genus and species level. The microbial profiles from soil and water, alongside distinctive microbial communities found in specific environments (such as urban versus rural areas, or forests versus deserts), can significantly aid in determining the location of death. Additionally, integrating microbiome data with other environmental factors—like temperature, humidity, and regional vegetation—enhances the accuracy of these determinations.

A corpse in a distinct environment might assimilate specific microbial communities, identifiable through high-throughput sequencing (Fierer and Jackson, 2006). Pechal et al. (2014) proposed that varying soil types, each with its unique microbial attributes, could be used to infer if a body was relocated after death, indicates that the microbial profile of a corpse is influenced by its immediate environment. Techniques like CNN are adept at extracting pivotal features from microbiome data, potentially tying them to the environment of the corpse. Researchers compared the diatom populations in the lung tissue of drowning rats with the diatom profiles in different sites of rivers established through CNN models, in order to infer the location of drowning (Zhang et al., 2021). By training a deep learning model to identify microbial patterns from specific environments, one can predict the likely location of the body based on its microbial composition. For instance, if a model is adept at distinguishing between microbial patterns of forests and deserts, it could potentially predict whether a body was located in a forest or a desert based on its microbial profile (Knights et al., 2011a). The researchers established a prediction model based on deep learning approach (Long Short Term Memory (LSTM)) by integrating weather variables and seasonal factors, which achieved higher performance in predicting microbial quality in drinking water (Mohammed et al., 2021). Although in its infancy, preliminary studies suggest that microbiome data can provide insightful leads about the place of death.

## Conclusion

AI models significantly enhance traditional microbiome analysis by adeptly managing the variability and intricacies of microbial communities. They also reduce errors in sequence alignment and classification. In forensic microbiology, particularly, deep learning has revolutionized the processing of high-throughput microbiome data. These AI models are not only efficient in handling large volumes of data but have also notably improved the precision in estimating the PMI and crime location. Machine learning models, such as random forests and support vector machines, are instrumental in predicting microbial functional pathways. This capability provides valuable forensic insights into the lifestyle, health, or environmental exposures of the deceased. Furthermore, AI’s proficiency in integrating data from various fields—metagenomics, transcriptomics, and proteomics—offers a comprehensive understanding of microbial functionalities in forensic contexts.

However, this field still faces significant challenges that need addressing. Precise AI models necessitate a substantial amount of high-quality, diverse microbiome samples, each meticulously annotated with environmental data. Although AI models demonstrate high accuracy rates, their internal mechanisms often remain opaque. The development of more interpretable models is critical, as they need to elucidate their predictive processes and decisions—particularly important in forensic contexts where the rationale behind conclusions must be clear and justifiable. Moreover, AI models must undergo rigorous validation under diverse conditions to confirm their broad applicability in various forensic scenarios. The current lack of standardization and repeatability in AI-driven microbiome analysis in forensics poses a considerable challenge. To overcome this, the development of standardized protocols is essential, alongside ensuring uniformity in data collection and processing. As the intersection of forensic microbiology and AI continues to progress, addressing these challenges will be vital for the reliable and effective application of these technologies in forensic science.

## Author contributions

ZW: Writing – original draft. YG: Writing – original draft. MH: Writing – review & editing. WY: Writing – review & editing. YL: Writing – review & editing. JM: Writing – review & editing. LL: Writing – review & editing. CL: Writing – review & editing. YiL: Writing – review & editing. JN: Writing – review & editing.
